# Analysis of DNA methylation changes following Cfp1 knockout in mouse spermatocytes

**DOI:** 10.5713/ab.24.0807

**Published:** 2025-02-27

**Authors:** Chanhyeok Park, Youngsok Choi, Seonho Yoo, Hyeonwoo La, Kwonho Hong

**Affiliations:** 1 Department of Stem Cell and Regenerative Biotechnology, Institute of Advanced Regenerative Science, Konkuk University, Seoul, Korea

**Keywords:** CFP1, DNA Methylation, Reduced-representation Bisulfite Sequencing, Spermatocytes

## Abstract

**Objective:**

Spermatogenesis is a complex biological process that encompasses meiosis in spermatocytes and the dynamic epigenetic alterations that ensure the inheritance of genetic traits. CXXC finger protein 1 (*CFP1, Cfp1, CXXC1, Cxxc1*) is a critical component of the SET domain-containing 1A histone lysine methyltransferase complex that catalyzes histone H3K4 methylation and has a specific binding domain for unmethylated CpG DNA. However, our current understanding of CFP1’s role in the genome-wide regulation of DNA and H3K4 methylation remains limited.

**Methods:**

We performed genome-wide methylation analysis using reduced-representation bisulfite sequencing on spermatocytes isolated from *Cfp1* knockout and wild-type mice. Promoter methylation changes were integrated with publicly available microarray and ChIP-seq data to identify genes regulated by CFP1.

**Results:**

CFP1 depletion led to significant alterations in DNA methylation, particularly in promoter regions of genes associated with meiosis, transcription regulation, and chromatin remodeling. A total of 21 genes were identified as direct targets of CFP1, exhibiting reduced promoter methylation and CFP1 binding.

**Conclusion:**

Our study findings contribute to elucidating the regulatory mechanisms of CFP1 in spermatocytes, providing valuable insights into the reproductive process and advancing our understanding of the underlying causes of infertility.

## INTRODUCTION

Spermatogenesis is a highly complex process involving the unique division of germ cells through meiosis, resulting in the production of haploid sperm [1]. Meiosis is characterized by dynamic chromosomal changes leading to homologous recombination, which plays an important role in generating genomic diversity [2]. Epigenetic changes are accompanied by complex molecular mechanisms. During spermatogenesis, dynamic changes in DNA methylation occur passively during the meiotic S phase [3]. These changes result in DNA hemimethylation during early meiotic prophase. DNA methylation levels were 25% lower in pachytene spermatocytes than in the spermatogonia, indicating a decrease in methylation during this stage [4]. Cytosine demethylation of male germ cell-specific genes is essential to initiate meiosis. Additionally, ubiquitin-like with PHD and ring finger domains 1 (UHRF1) may repress the hydroxylation of 5′-methylcytosine to 5′-hydroxymethylcytosine (5hmC) during male meiotic prophase. UHRF1 deficiency increases 5hmC levels by elevating the expression of the TET methylcytosine dioxygenase 1 transcription co-activator and altering gene expression in meiotic spermatocytes [5]. The activity of DNA methyltransferases (DNMTs) changes significantly during spermatogenesis, with reduced DNA methylation observed in type B spermatogonia and preleptotene spermatocytes, which are important for meiotic initiation [6]. *DNMT3L*, a member of the *DNMT3* family, has been identified as a regulator of the meiotic process because a reduction in its levels can lead to the early initiation of meiosis [7]. In the context of gene expression regulation, CpG islands located in gene promoters involved in development and essential cellular functions remain unmethylated, enabling their activation and proper expression [8]. Of all the histone modifications, H3K4 methylation is particularly increased near gene promoters, facilitating the recruitment of translation machinery and promoting transcriptional activation [9]. Additionally, dynamic changes in H3K4me2/3 modifications during spermatogenesis regulate chromatin structure and gene expression, ultimately playing an important role in the generation of haploid sperm [10]. H3K4me3 levels increase during the prophase stage of the first meiotic process. This dynamic change in H3K4me3 levels is associated with the activation of gene expression and plays a crucial role in regulating the progression of meiosis [11]. During spermatogenesis, the interplay between DNA methylation and histone modifications, including H3K4me, plays a critical role in the fine-tuning of gene expression to ensure the accessibility and activity of key genes.

CpG binding protein 1 (CFP1) binds to nonmethylated CpG islands and facilitates the deposition of H3K4me3 via the SET1 complex. Recently, CFP1 has emerged as a crucial player in the modulation of chromatin structure during meiosis [12–15]. CFP1 functions as an integral component of the SET1 complex (a methyltransferase complex responsible for H3K4 methylation), and facilitates the recruitment of the SET1 complex to the CpG islands of active genes, thereby promoting H3K4 methylation [10,16–18]. However, its function in the regulation of DNA methylation during meiosis remains unclear.

In this study, we examined the role of CFP1 in the genome-wide regulation of DNA methylation during meiosis using conditional *Cfp1* knockout (KO) spermatocytes. *Cfp1* deletion leads to widespread changes in DNA methylation patterns, particularly in the promoter regions of genes that are vital for meiosis and spermatocyte development. These findings underscore the importance of CFP1-mediated methylation in maintaining proper gene expression during spermatogenesis and suggest that disruptions in CFP1 function may have significant implications for male fertility.

## MATERIALS AND METHODS

### Animal care and generation of conditional KO mice

The mice used in this study were housed and maintained at the Konkuk University Animal Center in Seoul, Korea. *Cfp1*^F/F^ transgenic mice were generously provided by Dr. Skalnik of Indiana University. Transgenic (Stra8-icre) mice (Stock Number: 008208) were obtained from Jackson Laboratory (Bar Harbor, MA, USA) and crossed with *Cfp1*^F/F^ mice to generate *Cfp1*^F/+^; *Stra8*-icre and *Cfp1*^F/F^; *Stra8*-icre (*Cfp1* KO, *Cfp1*^Stra8^) mice. All animal care and experimental procedures followed the guidelines outlined in the Guide for the Care and Use of Laboratory Animals, and were approved by the Institutional Animal Care and Use Committee of Konkuk University (IACUC approval number: KU22181).

### Isolation of spermatocytes

Pooled spermatocytes from P21 mice were used for the sequencing experiments. Seminiferous tubules from C57BL/6 and *Cfp1*^Stra8^ mice were collected and minced. The minced tissue was incubated with Krebs-Ringer bicarbonate medium (EKRB) containing 0.5 mg/mL collagenase at 33°C for 1 h to facilitate dissociation. The dissociated seminiferous tubules were further digested with EKRB supplemented with 0.5 mg/mL trypsin and 1 μg/mL DNase I at 33°C for 15 min. Spermatocytes were isolated using the sedimentation velocity method at unit gravity and a temperature of 5°C [19]. Isolated cells were suspended in EKRB containing 0.5% bovine serum albumin and placed in a sedimentation chamber at a flow rate of 10 mL/min. The purified spermatocytes were lysed for subsequent sequencing. Using this method, at least 80% of the spermatocytes exhibiting SYCP1/SYCP3 immunostaining were consistently observed in the testes of 3-week-old mice.

### Preparation of a reduced-representation bisulfite sequencing library

The existing single-enzyme reduced-representation bisulfite sequencing (RRBS) (sRRBS) method, which utilizes the MspI restriction endonuclease, can be enhanced by incorporating an additional enzyme, ApeKI [20]. Genomic DNA (500 ng) was digested with Msp1 (NEB, Ipswich, MA, USA) at 37°C for 7 h and then with ApeKI (NEB) at 75°C for 16 h. The digested DNA fragments were purified using a MiniElute PCR Purification kit (Qiagen, Valencia, CA, USA). End repair was performed using a repair solution containing T4 DNA polymerase (NEB), Klenow fragment, T4 polynucleotide kinase (NEB), dNTPs, and 1X polynucleotide kinase buffer at 20°C for 30 min. Subsequently, A-tailing was performed using Klenow (3′ → 5′ exo-; NEB) at 37°C for 30 min. DNA was purified using a MiniElute PCR Purification kit (Qiagen). The purified DNA fragments were ligated to an adapter (Illumina, San Diego, CA, USA) using T4 DNA ligase at 65°C for 15 min, followed by 20°C for 15 min. DNA fragments of 160 to 240 bp were selected by electrophoresis on a 2% agarose gel. Bisulfite conversion was performed using a Methylation Gold kit (ZYMO RESEARCH, Tustin, CA, USA) according to the manufacturer’s instructions. Polymerase chain reaction (PCR) amplification of bisulfite-converted products was performed using Pfu Turbo Cx hotstart DNA polymerase (Agilent Technologies, Santa Clara, CA, USA) with the supplied primers (forward: 5′-AATGATACGGCGACCACCGAGAT-3′, reverse: 5′-CAAGCAGAAGACGGCATACGA-3′). PCR conditions included an initial denaturation at 94°C for 1 min, followed by 11 to 15 cycles of denaturation at 94°C for 10 s, annealing at 58°C for 30 s, extension at 72°C for 30 s, and a final extension at 72°C for 5 min. The resulting library was analyzed using an Agilent 2100 Bioanalyzer (Agilent Technologies) and quantified using real-time PCR. RRBS libraries were generated using the TruSeq Standard Genomic DNA Sample Preparation kit (Illumina) according to the manufacturer’s instructions.

### Computational processing of the sequencing data

The single-cell RNA sequencing (scRNA-seq) dataset GSE120508 was reanalyzed to examine *CXXC1* expression across various cell types in the human testis [21]. For RRBS data analysis, sequencing reads were aligned to the mm10 genome using Bismark (v0.16.3) [22] and Bowtie (v2.2.9) [23], and the total number of mapped reads for each sample was determined using SAMtools idxstats [24]. The --non_directional option was used to allow alignment of bisulfite-converted reads without strand bias, and the resulting BAM files were sorted and indexed using SAMtools. DNA methylation ratios at CpG sites were extracted from the aligned reads using the bismark_methylation_extractor tool with the --comprehensive and --bedGraph options, producing methylation summaries in both CX report and BEDgraph formats. To ensure high data quality, CpG sites with insufficient coverage (<10x) were excluded from downstream analysis. Deeptools (v.3.1.3) was used to analyze the mapped read data using the Spearman correlation heatmap and to assess the principal component analysis (PCA) results [25,26]. To assess promoter region methylation, we employed the Bedtools Multicov tool, which calculates the number of reads covering each genomic interval defined in the BED file [27]. For each region, we calculated the reads per kilobase of transcript per million mapped reads (RPKM) using the following formula (C: number of mapped reads within the genomic region, L: length of the region, and N: total number of mapped reads for the sample) [28,29]:


$$RPKM=\frac{10^{9}*C}{\left(L*N\right)}$$

Hierarchical clustering analysis of global methylation levels across different samples was performed using the heatmap.2 function from the gplots package in R, allowing visualization and comparison of methylation patterns across various genomic regions. Additionally, the genomic distribution of key methylation markers was examined using Integrative Genomics Viewer (IGV), providing a detailed visualization of their positioning and methylation status across the genome.

### Comparative analysis of DNA methylation with public dataset

To identify genes with CFP1 binding and altered expression, we utilized publicly available microarray and CFP1 ChIP-seq data from the NCBI Gene Expression Omnibus (GEO) under accession numbers GSE121240 (microarray) and GSE120994 (CFP1 ChIP-seq). The analytical methods for these datasets were adapted from previously reported methods. Additionally, RRBS data were deposited in the GEO under accession number GSE240280 (RRBS).

## RESULTS

### CFP1 as a potential regulator in meiosis

**Figure 1:**
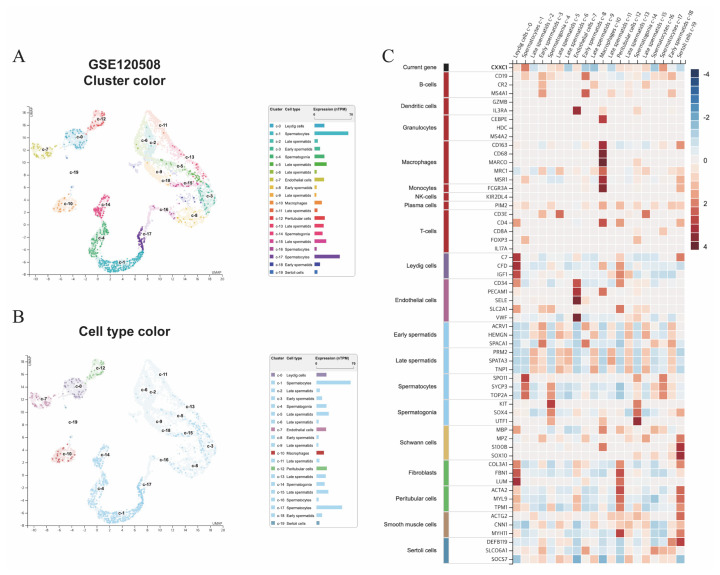
Single-cell transcriptome analysis of whole testis in human, highlighting *CXXC1* expression in spermatocytes. (A) Uniform Manifold Approximation and Projection (UMAP) plot showing clustering of scRNA-seq data from whole testis samples, colored by cluster identity. (B) UMAP plot of the same scRNA-seq data, colored by cell type identity as determined by marker gene expression. (C) Heatmap displaying the expression levels of selected marker genes across various single-cell types.

To investigate the expression pattern of *Cxxc1* in different cell types in the mouse testis, we reanalyzed a publicly available scRNA-seq dataset (GSE120508) [21]. The dataset comprises a comprehensive single-cell transcriptome of the whole testis, allowing for a detailed cell type-specific expression analysis. Using Uniform Manifold Approximation and Projection (UMAP) for dimensionality reduction, distinct clusters corresponding to various testicular cell populations were identified (Figure 1A). Each cluster was annotated based on known marker genes, revealing a complex cellular composition including spermatogonia, spermatocytes, Sertoli cells, Leydig cells, and various immune cell types. Further analysis colored the UMAP plots according to cell type identity (Figure 1B), enabling the visualization of specific cell types within each cluster. Our results indicate that *Cxxc1* expression is predominantly elevated in spermatocytes. This observation was reinforced by a heatmap analysis (Figure 1C), which showed that *Cxxc1* is one of the most highly expressed genes in spermatocytes compared to other cell types.

### CFP1 ablation alters DNA methylation levels in spermatocytes

**Figure 2:**
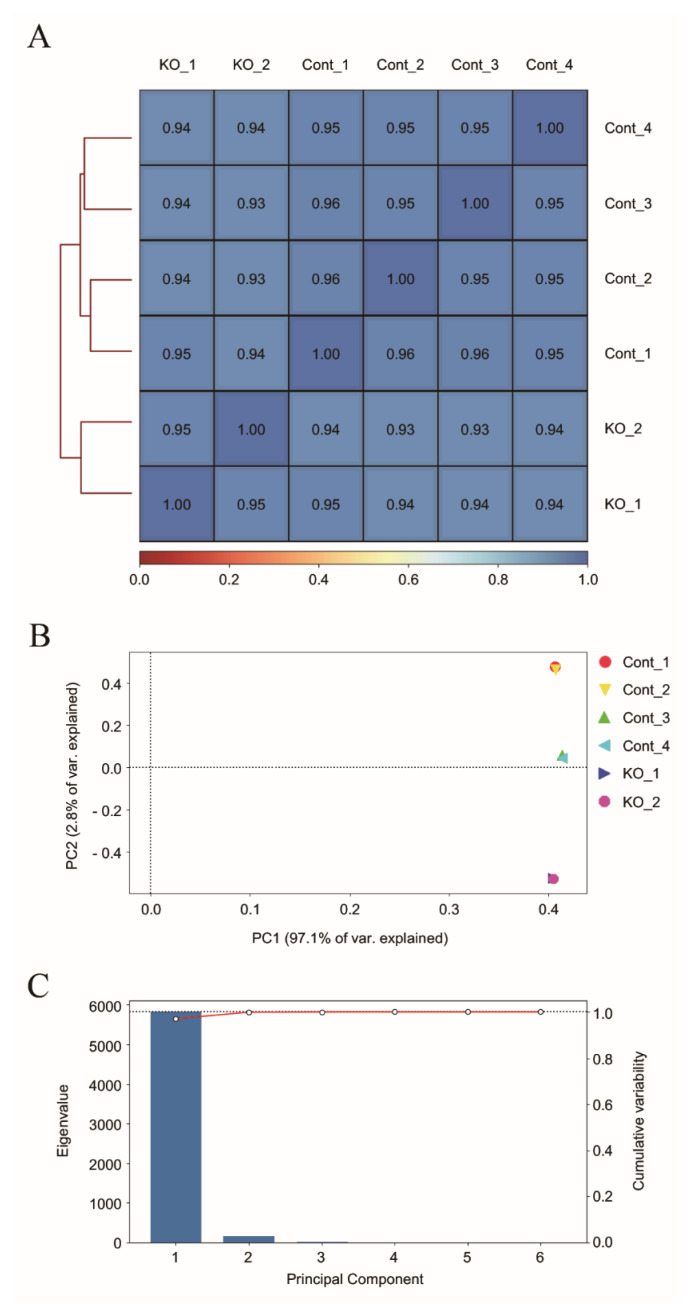
Comparative genomic analysis of *Cfp1* knockout (KO) and wild type (WT) cells. (A) Spearman correlation heatmap showing high intragroup correlations between KO and WT samples, with clear separation between groups. (B) PCA plot displaying clustering of KO and WT samples along the first two principal components, reflecting differences in methylation patterns. (C) Scree plot illustrating that the first principal component explains most of the variance, confirming effective dimensionality reduction. PC, principal component; PCA, principal component analysis.

To investigate the genomic impact of the *Cfp1* KO in mouse cells, data from RRBS were analyzed. Both *Cfp1* KO and wild type (WT) samples were compared to uncover differences in methylation profiles between the two conditions. The Spearman correlation heatmap (Figure 2A) illustrates the relationships between read counts from the RRBS datasets across KO and WT cells. Notably, samples from both *Cfp1* KO and WT cells showed strong intragroup correlations, indicating consistent methylation patterns within each condition. Hierarchical clustering further validated these results as the KO and WT samples clustered separately, highlighting the distinct methylation profiles associated with *Cfp1* deletions. The PCA plot (Figure 2B) revealed the separation of *Cfp1* KO and WT samples along the first two principal components (PC1 and PC2), which together explained a significant portion of the variance in the methylation data. The distinct clustering of *Cfp1* KO and WT samples reflected the substantial impact of *Cfp1* deletion on genomic methylation patterns. A scree plot (Figure 2C) indicated that the first few principal components captured most of the variance in the data, confirming the effectiveness of PCA for dimensionality reduction. Coverage analysis revealed a consistent sequencing depth across the KO and WT conditions in the RRBS data, confirming the robustness and reliability of the methylation data used in this study.

### Altered methylation levels in gene promoters are associated with spermatogenesis

**Figure 3:**
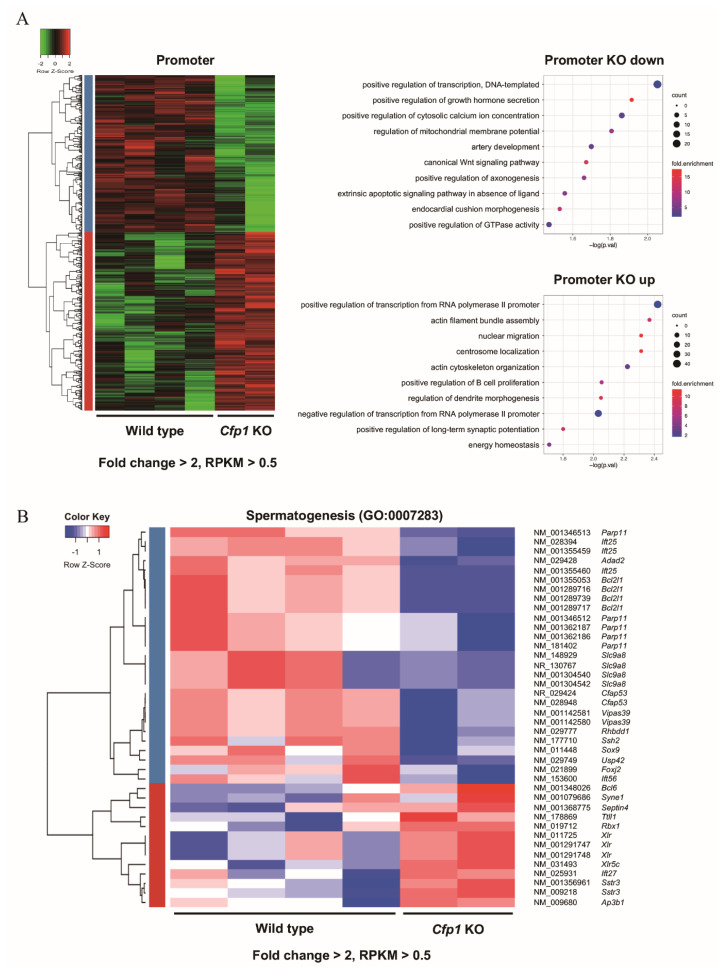
Differential methylation in promoter regions of *Cfp1* knockout cells. (A) Heatmap depicting promoter methylation changes between wild type (WT) and *Cfp1* knockout (KO) cells. (B) Heatmap displaying differential methylation of spermatogenesis-related genes (GO:0007283) between WT and KO cells. RPKM, reads per kilobase of transcript per million mapped reads; GO, Gene Ontology.

To explore the genome-wide methylation changes resulting from *Cfp1* KO, we quantified the methylation levels in the promoter and gene body regions, expressed as RPKM. A heatmap revealed significant differences in promoter methylation between WT and *Cfp1* KO cells (Figure 3A). Promoter regions in *Cfp1* KO cells showed substantial alterations, with numerous regions exhibiting either increased or decreased methylation compared with those in WT cells. The top Gene Ontology (GO) terms for genes with downregulated promoter methylation in *Cfp1* KO cells included processes such as “positive regulation of transcription” and “positive regulation of growth hormone secretion”. For genes with upregulated promoter methylation, the most enriched GO terms were related to the positive regulation of transcription from RNA and actin filament bundle assembly. Methylation levels in the gene body regions showed significant changes in *Cfp1* KO cells relative to those in WT cells (Supplement 1). Furthermore, we observed changes in the promoter methylation of genes associated with spermatogenesis (Figure 3B). Our analysis identified 40 spermatogenesis-related genes, including transcript variants with altered methylation patterns. These findings underscore the extensive impact of *Cfp1* deletion on epigenetic regulation, affecting a wide range of biological processes, and potentially influencing cellular functions and phenotypic outcomes.

### CFP1 regulates meiotic gene expression by modulating DNA methylation

**Figure 4:**
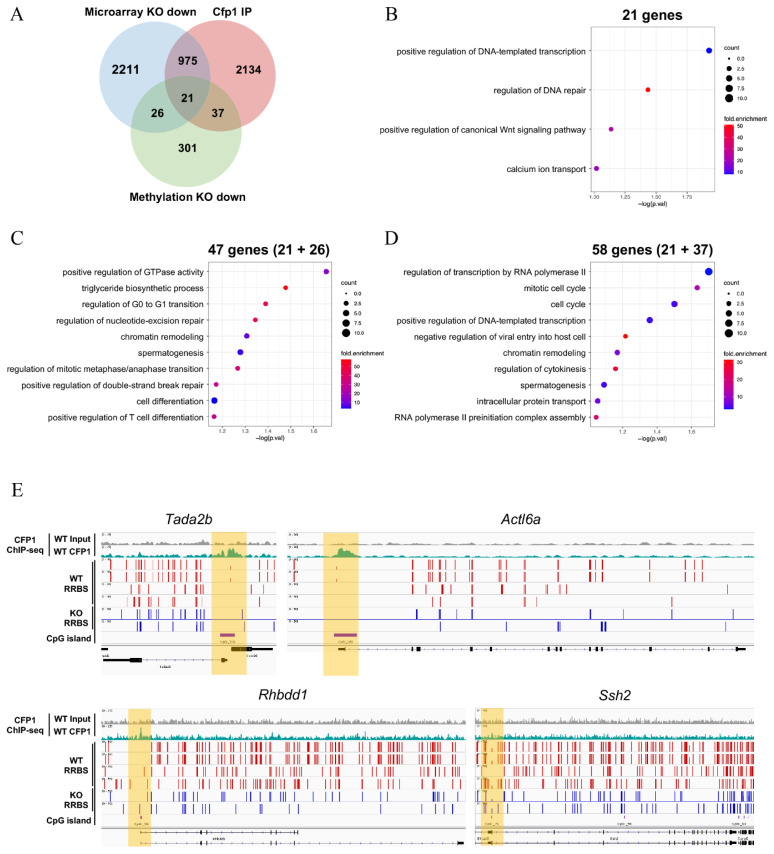
Integration of epigenetic and transcriptional data in *Cfp1* knockout cells. (A) Venn diagram showing the overlap between three datasets: genes downregulated in *Cfp1* knockout (KO) mice, CFP1-bound genes, and genes with reduced promoter methylation. (B) Gene ontology (GO) analysis of the 21 overlapping genes associated with transcriptional and genomic regulatory processes. (C) GO analysis of 47 genes that are downregulated in the microarray but not bound by CFP1. (D) GO analysis of 58 genes bound by CFP1 and showing reduced promoter methylation. (E) Integrative Genome Viewer tracks showing methylation and CFP1 binding patterns for selected genes (*Tada2b*, *Actl6a*, *Rhbdd1*, and *Ssh2*). WT, wild type.

To analyze genome-wide epigenetic and transcriptional changes associated with *Cfp1* KO, we integrated data from a previous study [15] and conducted a comprehensive investigation. A constructed Venn diagram (Figure 4A) illustrates the overlap among the three datasets: genes downregulated in *Cfp1* KO mice from microarray analysis (Microarray KO down), genes bound by CFP1 (CFP1 IP), and genes exhibiting reduced methylation in promoter regions from RRBS (methylation KO down). Notably, 21 genes overlapped across all three datasets, suggesting that they are direct targets of *Cfp1* whose transcription is regulated by both DNA methylation and CFP1 binding. GO analysis of these 21 overlapping genes (Figure 4B) highlights two key biological processes (“positive regulation of DNA-templated transcription” and “regulation of DNA repair”), indicating potential roles for these genes in maintaining genomic stability and transcriptional regulation. We further examined the 47 genes that were downregulated in the microarray analysis and exhibited reduced promoter methylation in *Cfp1* KO cells, but were not bound by CFP1 (Figure 4C; 47 genes; 21+26). GO analysis of this subset revealed enrichment for processes such as “positive regulation of GTPase activity,” “spermatogenesis,” and “chromatin remodeling.” These results suggest that altered methylation patterns in these genes contribute to changes in chromatin structure and cellular processes that are critical for spermatogenesis. Finally, we performed GO analysis on the 58 genes that were bound by CFP1 and exhibited reduced promoter methylation in *Cfp1* KO cells (Figure 4D; 58 genes; 21+37). The most significantly enriched processes for this group included “regulation of transcription by RNA polymerase II,” “mitotic cell cycle,” and “chromatin remodeling.” This suggests that CFP1 binding plays a critical role in regulating genes involved in these fundamental biological processes, potentially through its influence on promoter methylation. In addition to GO analysis, we utilized IGV to further explore the genomic distribution of the key genes identified through GO analysis (Figure 4E). This visualization allowed us to examine the methylation and binding patterns of specific genes enriched in key biological processes. For example, the tracks for *Tada2b, Actl6a, Rhbdd1*, and *Ssh2* show how methylation changes in the promoter regions are reflected across different conditions. These genes, which are involved in transcriptional regulation, chromatin remodeling, and spermatogenesis, were selected based on their significant enrichment in GO analyses.

## DISCUSSION

Our study sheds new light on the critical role of Cfp1 in spermatogenesis, particularly in regulating meiotic gene expression via epigenetic mechanisms. Consistent with previous findings underscoring the importance of Cfp1 in meiotic progression via transcriptional regulation and H3K4 methylation at promoters [9,14,15,30], our reanalysis of publicly available single-cell RNA sequencing data from human testes revealed that *CXXC1*, the gene encoding CFP1, was predominantly elevated in spermatocytes [21]. This suggests that CFP1 plays a vital role in the meiotic phase of germ cell development (Figure 1).

Recent research has underscored the pivotal role of aberrant DNA methylation in sperm abnormalities, particularly in patients with conditions such as asthenospermia and oligoasthenospermia [31]. These findings highlight the importance of epigenetic mechanisms that contribute to male infertility by influencing normal sperm development. DNA methylation is involved in pathological conditions and is also essential for normal spermatocyte development. Genome-wide methylation analyses of testicular germ cells from men with normal spermatogenesis have revealed significant patterns, notably a global reduction in DNA methylation in primary spermatocytes, which are characteristic of meiotic cells [32,33]. This hypomethylation likely facilitates chromatin remodeling and changes in the gene expression required for meiotic progression.

Building on this understanding, we investigated the effects of *Cfp1* KO on spermatocytes, where *Cfp1* is normally highly expressed, to examine genome-wide methylation changes. Our RRBS data demonstrated distinct methylation profiles between *Cfp1* KO and WT cells, with significant alterations in promoter methylation patterns observed in the absence of *Cfp1* (Figures 2, 3). The high intragroup correlations and clear separation in PCA plots between KO and WT samples indicate that *Cfp1* deletion leads to substantial changes in genomic methylation patterns, aligning with the notion that CFP1 functions as a chromatin regulator by depositing H3K4me3 at the promoter regions.

Importantly, differential methylation in the promoter regions of *Cfp1* KO cells highlighted significant epigenetic alterations in genes associated with spermatogenesis (Figure 3). GO analysis revealed that genes with altered promoter methylation were enriched in processes that included positive regulation of transcription, chromatin remodeling, and actin filament bundle assembly. Specifically, we identified 40 spermatogenesis-related genes that exhibited altered methylation patterns, underscoring the extensive impact of *Cfp1* deletion on epigenetic regulation and its potential influence on spermatogenic failure.

Our integrated analysis of epigenetic and transcriptional data provides compelling evidence that *Cfp1* directly regulates a subset of genes critical for meiosis and spermatogenesis. The Venn diagram illustrates the overlap between the genes downregulated in *Cfp1* KO mice, those bound by CFP1, and those with reduced promoter methylation (Figure 4A). The 21 genes that overlapped across all datasets were likely direct targets of *Cfp1*, whose transcription is regulated through both DNA methylation and direct binding. GO analysis of these overlapping genes highlighted key biological processes, such as positive regulation of DNA-templated transcription and regulation of DNA repair (Figure 4B), which are both essential for meiotic progression and maintenance of genomic integrity.

Our research revealed no significant difference in gene expression levels; *Rhbdd1* and *Ssh2*, whose methylation decreased due to CFP1 binding, played crucial roles in spermatogenesis (Figures 4D, 4E). According to a recent study, *Rhbdd1* is essential for survival and differentiation of spermatogonia during mammalian spermatogenesis [34]. The GC-1 spermatogonia cell line featuring downregulated mRHBDD1 display increased sensitivity to apoptotic stimuli and are unable to survive and differentiate within the seminiferous tubules. Similarly, according to recent research, *Ssh2* is vital for acrosome formation and male fertility through actin remodeling [35]. SSH2-deficient mice exhibit arrested spermatogenesis at the early spermatid stage, increased apoptosis, and impaired acrosome formation due to disrupted transport and fusion of proacrosomal vesicles. Additionally, these mice showed disorganized F-actin structures resulting from the excessive phosphorylation of COFILIN. The involvement of CFP1 in these genes and the observed methylation changes implied that *Cfp1*-mediated methylation is important for spermatogenesis at precise cellular stages, suggesting significant implications for future research.

Moreover, our findings highlight that the role of *Cfp1* extends beyond more chromatin modification; it also acts as a guardian of the meiotic process by orchestrating the expression of spermatogenesis-related factors. Altered methylation patterns and transcriptional changes observed in genes involved in chromatin remodeling and cell cycle regulation suggest that the absence of *Cfp1* disrupts the delicate balance of gene expression required for successful spermatogenesis [36]. Furthermore, reanalysis of publicly available data from other mammalian models revealed that *Cfp1* expression is significantly elevated in pachytene spermatocytes (data not shown). This finding supports the notion that CFP1 plays a conserved and critical role in male germ cell development across species.

In conclusion, our comprehensive analysis underscores the essential role of *Cfp1* in the epigenetic and transcriptional regulation of spermatogenesis. Loss of *Cfp1* disrupts promoter methylation patterns and alters the expression of genes crucial for meiotic progression and chromatin remodeling. These results deepen our understanding of complex epigenetic regulation in germ cells and shed light on the contribution of genetic and epigenetic factors to male infertility. Future studies should focus on elucidating the detailed mechanisms by which CFP1 interacts with other chromatin modifiers and transcription factors, as well as exploring potential therapeutic targets for treating male idiopathic azoospermia.

## SUPPLEMENTARY MATERIAL

**Supplement 1**. Differential methylation in gene body regions of Cfp1 knockout cells.
