# Supplementary material for: Analysis of DNA methylation changes following Cfp1 knockout in mouse spermatocytes
Source: Anim Biosci. 2025 Feb 27;38(8):1570–9. doi: 10.5713/ab.24.0807 (PMC12229914; doi:10.5713/ab.24.0807)
Supplement: Supplementary file 1 [file ab-24-0807-Supplementary-1.pdf]

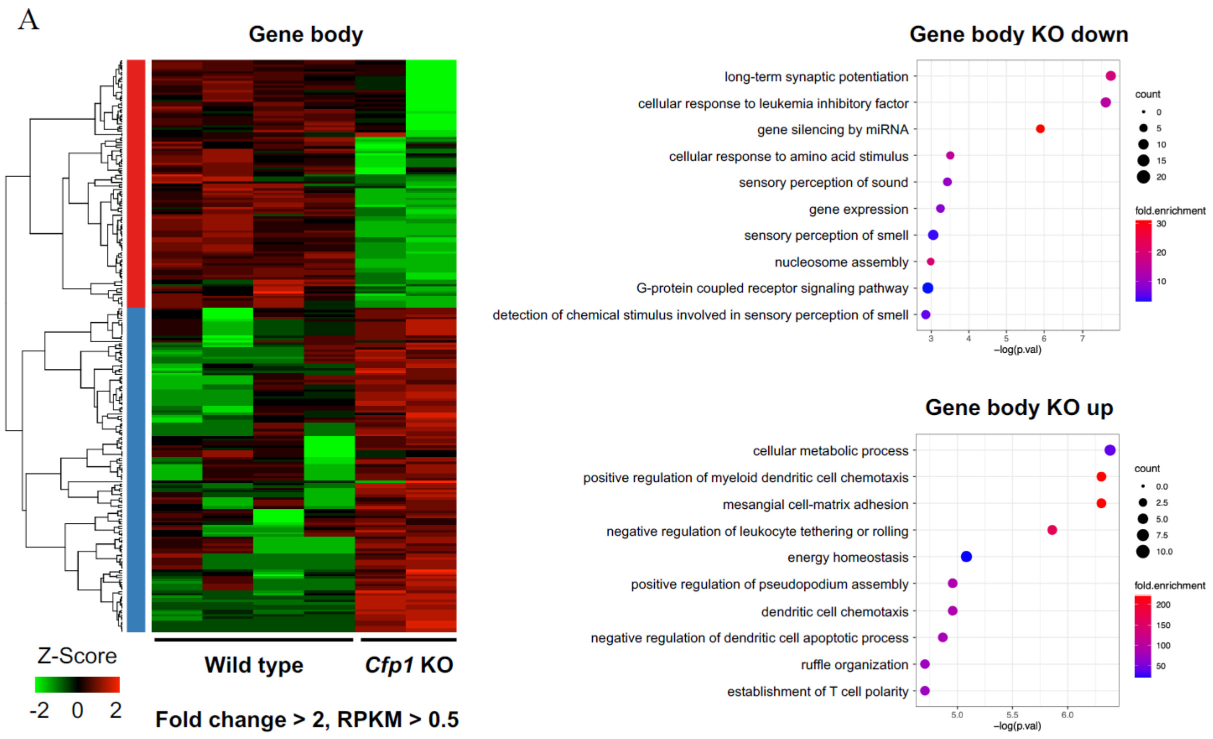

Supplement 1. Differential Methylation in Gene Body Regions of Cfp1 Knockout Cells. (A) Heatmap depicting gene body methylation changes between wild type (WT) and Cfp1 knockout (KO) cells.
